# Gender Differences in Health Expectancies across the Disablement Process among Older Thais

**DOI:** 10.1371/journal.pone.0121310

**Published:** 2015-03-23

**Authors:** Benjawan Apinonkul, Kusol Soonthorndhada, Patama Vapattanawong, Wichai Aekplakorn, Carol Jagger

**Affiliations:** 1 Institute for Population and Social Research, Mahidol University, Salaya, Phutthamonthon, NakhonPathom, Thailand; 2 Department of Community Medicine, Faculty of Medicine, Ramathibodi Hospital, Mahidol University, Bangkok, Thailand; 3 Newcastle University Institute for Ageing and Institute of Health and Society, Newcastle University, Newcastle upon Tyne, United Kingdom; School of Public Health of University of São Paulo, BRAZIL

## Abstract

**Objectives:**

To estimate health expectancies based on measures that more fully cover the stages in the disablement process for the older Thais and examine gender differences in these health expectancies.

**Methods:**

Health expectancies by genders using Sullivan’s method were computed from the fourth Thai National Health Examination Survey conducted in 2009. A total of 9,210 participants aged 60 years and older were included in the analysis. Health measures included chronic diseases; cognitive impairment; depression; disability in instrumental activities of daily living (IADL); and disability in activities of daily living (ADL).

**Results:**

The average number of years lived with and without morbidity and disability as measured by multiple dimensions of health varied and gender differences were not consistent across measures. At age 60, males could expect to live the most years on average free of depression (18.6 years) and ADL disability (18.6 years) and the least years free of chronic diseases (9.1 years). Females, on the contrary, could expect to live the most years free of ADL disability (21.7 years) and the least years free of IADL disability (8.1 years), and they consistently spent more years with all forms of morbidity and disability. Finally, and for both genders, years lived with cognitive impairment, depression and ADL disability were almost constant with increasing age.

**Conclusion:**

This study adds knowledge of gender differences in healthy life expectancy in the older Thai population using a wider spectrum of health which provides useful information to diverse policy audiences.

## Introduction

During the last three decades of the twentieth century Thailand’s population has aged as a result of the demographic transition from levels of high to low mortality and fertility [1]. In 1970 older people formed only five percent of the total population [2], but by 2014 this had risen to 15 percent [3] and is projected to reach 32 percent by 2040 [4]. As a consequence of falling mortality, life expectancy at birth of the Thai population has risen continuously, from 60 years in 1967 [1] to 71 years for males and 78 years for females in 2014 [3]. Thai life expectancy at birth now ranks the fourth highest for males and the third highest for females in the ten Association of Southeast Asian Nations (ASEAN) countries [5]. Moreover projections estimate that Thai life expectancy at birth will reach 75 years for males and 82 years for females by 2040 [4].

Longer life expectancy and the concomitant increase in the occurrence of non-communicable diseases have raised a question of the quality of years lived. Health expectancy is an index of population health, representing the increasing focus on quality of life lived rather than on the quantity as measured by life expectancy [6,7]. Health expectancy combines information on mortality from life tables with epidemiologic data on morbidity and disability prevalence [7]. It is composed of estimated lengths of time spent in different health states until death [8].

Basically, health expectancies are the combination of life expectancy with health concepts, which make it possible to calculate years lived in different states of health [6,8]. One of the most appropriate frameworks to elucidate more detailed health measures for the older population is the disablement process [9]. Models of the disablement process identify pathologies or diseases as the earliest event, leading to dysfunctions in body systems (physical or mental), restriction in physical or mental actions, and finally limitations in doing activities in any domain of life [9]. Along this pathway, there are personal factors (e.g. lifestyle and behaviour changes, activity accommodation) and factors external to the individuals (e.g. medical care and rehabilitation, environmental factors) operating to alter the passage between stages. Though this model is more ‘linear’, it is in concordance with the World Health Organization International Classification of Functioning, disability and Health (ICF) [10].

Health expectancies have been computed for a number of countries based on several health measures [8,11]. Most studies in Asian countries have concentrated on disability rather than earlier stages in the chain of disablement process and on a single health measure [11]. In Thailand estimates have been made of years with self-care disability [13–15], mobility disability [15], and in different states of perceived health [16] at older ages with results varying by the measure used and the time period of the study. Regardless of the underlying health measure, gender differences in the Thai population have consistently shown longer life but more years in poor health for females than males.

Nevertheless, estimates of health expectancies which cover the spectrum of the disablement process remain unknown, particularly for the Thai population. Estimates of health expectancy in different states of health are useful for diverse policy audiences [17] and further, could provide information on the passage between health stages along the disablement pathway. This study therefore uses the most recent Thai national data from 2009 to estimate health expectancies that more fully cover the stages in the disablement process. More specifically the study estimates years of life at older ages with: chronic diseases; cognitive impairment; depression; disability in instrumental activities of daily living (IADL); and disability in activities of daily living (ADL), and examines gender differences in these health expectancies.

## Materials and Methods

### Data

Data on morbidity and disability were drawn from the fourth Thai National Health Examination Survey (NHES IV) in 2009. The NHES IV is a cross-sectional survey using stratified multi-stage sampling of the Thai population to yield nationally representative samples. The sampling method has been described elsewhere [18]. Briefly, the first stage of sampling was systematic selection of five provinces in each of the four regions. In the second stage, three to five districts were randomly selected for each selected province and Bangkok. The third stage involved systematic selection of 13–14 electoral units in municipality areas or villages in non-municipal areas for each district. The final stage comprised random selection of individuals aged 15 years and older by selected electoral units and villages, gender, and age group. Finally, a total of 20,450 individuals were obtained. The present study focused on 9,210 participants aged 60 years and older (95% response rate). Information on participants was collected through a variety of methods, including a face-to-face interview, functional tests, physical examinations, and laboratory tests with data quality assured by training before and supervision during fieldwork, as well as rigorous data checking. The NHES IV was approved by the Ethical Review Committee for Research in Human Subjects, Ministry of Public Health. And, all participants provided written informed consent.

Mortality data was drawn from the Thai vital registration system, this being the most important source of mortality data. Every vital event (births and deaths) is mandated by civil registration law to be registered at the district offices or municipality registrars. At the end of each year, counts are made of the number of births and deaths (and thereby the total population), and these figures are made publicly available.

### Health measures

The measures on which health expectancies were based included chronic diseases, cognitive impairment, depression, and disability in IADL and ADL. Full definitions are provided in Table 1.

**Table 1:** Definitions of health measures.

Health measures	Definitions
Chronic diseases	Having at least 1 of 2 diseases; hypertension (diagnosed based on systolic blood pressure ≥140 mmHg, or diastolic blood pressure ≥90 mmHg, or self-reports of being under antihypertensive) and diabetes mellitus (diagnosed based on fasting plasma glucose level ≥126 mg/dl, or self-reports of diagnosis from physicians and being under medical treatments)
Depression	Being diagnosed as depression based on the criterion of Diagnostic and Statistical Manual of Mental disorders, 4^th^ edition [19], or self-reports of diagnosis from physicians and being under medical treatments within the last 12 months
Cognitive impairments	Having scores of Mini-Mental State Examination Thai version-2002 less than the cut-off points determined by the Institute of geriatric medicine, Thailand (14 out of 23 points for illiterate; 17 out of 30 points for primary school levels; and 22 out of 30 points for higher levels) [20]
ADL disability	Being unable to do at least one of basic self-care activities without assistances, which include feeding, dressing, bathing, toileting, and transferring from beds or chairs
IADL disability	Being unable to do at least one of activities without assistances, which include using telephone, handling finances, responsibility for own medication, using transportation or driving, light housework (sweeping, gathering things, and make beds), and heavy housework (wiping, carrying, dipping up water)

### Statistical methods

Health expectancies were calculated by Sullivan’s method [21,22] which applies the age- and sex-specific prevalence of health to divide the number of person years lived in the given age interval (from a period life table) into years lived with and without health problems. Health expectancy calculated by Sullivan’s method is the average number of remaining years, at particular age, which an individual can expect to live healthy.

The first step was to calculate the period life tables, for men and women, from the age-specific death rates which required some adjustment in (i) the number of deaths (for unknown age of death and under-registration) and (ii) the age-specific death rates for the very old. Overall and age-specific percent completeness of death registration obtained from other reports [23–25] was applied as correction factors. The Coale-Kisker method [26] was employed to estimate age-specific death rates for those aged 80 years and older as the values were too low when calculated directly. This method assumes the declining rate of increase in mortality rates at very old ages.

As health expectancies are subject to sampling variation from the health surveys, we calculated variance and used z-statistics to test gender differences in health expectancies following established methods [21].

## Results

Participants aged 60 years and older in the NHES IV were a slight majority of females (51%). The mean age of participants was 69.4 years (SD = 6.9) for males and 69.6 years (SD = 7.1) for females, respectively.

### Prevalence of morbidity and disability

Table 2 presents the age- and sex-specific prevalence of morbidity and disability, as measured by each health state, for the Thai population aged 60 years and over in 2009. Overall, females had a higher prevalence of morbidity and disability compared to males of the same age. The prevalence of all ill-health states tended to increase with age in both genders. Most noticeable is that the prevalence of cognitive impairment and depression in females was almost twice that in males (Table 2).

**Table 2:** Prevalence^a^ (in percent) of morbidity and disability by age and genders in Thailand, 2009 (95% CIs in parentheses).

Age	Chronic diseases		Cognitive impairment		Depression		ADL disability		IADL disability	
(Years)	Males	Females	Males	Females	Males	Females	Males	Females	Males	Females
60–64	46.0(42.0–50.1)	48.8*(45.3–52.3)	4.9(3.6–6.2)	8.0*(6.2–9.8)	2.6(1.9–3.2)	6.2*(5.1–7.2)	0.8(0.4–1.2)	1.3(0.6–1.9)	23.9(20.4–27.4)	44.9*(38.8–51.1)
65–69	51.2(45.2–57.2)	55.2*(51.5–58.9)	6.4(4.4–8.5)	8.7*(7.4–10.1)	2.6(1.5–3.6)	5.0*(3.9–6.0)	1.1(0.5–1.7)	1.0(0.5–1.6)	30.7(24.8–36.4)	52.2*(46.2–58.1)
70–74	50.0(45.5–54.5)	59.0*(55.3–62.7)	9.4(7.1–11.7)	14.3*(12.2–16.4)	2.6(1.6–3.6)	6.9*(5.5–8.3)	4.0*(0.8–7.2)	2.1(1.3–2.9)	34.9(27.4–42.3)	65.5*(59.8–71.3)
75–79	60.3(55.1–65.6)	58.1(52.8–63.3)	12.2(9.4–15.0)	23.6*(20.2–27.0)	3.5(1.6–5.4)	6.5*(4.6–8.3)	2.6(1.6–3.6)	2.0(1.3–2.8)	47.8(42.0–53.5)	76.2*(70.7–81.7)
80+	57.6(51.4–63.9)	61.9(57.1–66.7)	22.1(19.2–30.0)	40.0*(36.2–43.7)	3.7(1.7–5.7)	7.1*(3.4–10.7)	5.3(3.7–6.9)	10.0*(7.6–12.5)	67(61.3–72.7)	81.1*(76.7–85.5)

^a^With sampling weighted

*Significantly higher than the other gender (two-proportion z-test, p<0.05)

Source: Author’s computations from the NHES IV.

### Life and health expectancy

At age 60, males and females could expect to live on average a further 19.1 and 22.6 years, respectively (Table 3). Females had longer total life expectancy than males at every age, with the gaps between females and males ranging from 3.5 years at age 60 to 1.5 years at age 80.

**Table 3:** Total life expectancy and number of years lived free of morbidity and disability at age 60, 70, and 80 by genders in Thailand, 2009.

	Males	Females
Age 60		
Total life expectancy	19.1	22.6
Years lived free of (95%CI)		
Chronic diseases	9.1 (8.8–9.4)	9.8 (9.4–10.1)*
Cognitive impairment	17.1 (16.9–17.3)	18.1 (17.8–18.4)*
Depression	18.6 (18.5–18.7)	21.1 (21.0–21.3)*
ADL disability	18.6 (18.5–18.7)	21.7 (21.6–22.0)*
IADL disability	11.6 (11.3–11.8)*	8.1 (7.8–8.4)
Age 70		
Total life expectancy	12.9	15.3
Years lived free of (95%CI)		
Chronic diseases	5.7 (5.4–6.0)	6.1 (5.8–6.5)
Cognitive impairment	11.0 (10.8–11.2)	11.0 (10.7–11.4)
Depression	12.5 (12.4–12.6)	14.3 (14.1–14.5)*
ADL disability	12.4 (12.3–12.5)	14.5 (14.3–14.6)*
IADL disability	6.4 (6.1–6.7)*	3.8 (3.5–4.1)
Age 80		
Total life expectancy	8.6	10.1
Years lived free of (95%CI)		
Chronic diseases	3.6 (3.3–4.0)	3.8 (3.4–4.3)
Cognitive impairment	6.7(6.4–7.0)*	6.1 (5.6–6.5)
Depression	8.3 (8.1–8.4)	9.4 (9.1–9.6)*
ADL disability	8.1 (8.0–8.3)	9.1 (8.8–9.3)*
IADL disability	2.8 (2.5–3.2)*	1.9 (1.6–2.3)

*Significantly higher than the other gender at 95% confidence intervals.

At age 60, males could expect to live the most years on average free of depression (18.6 years, 95%CI 18.5–18.7) and ADL disability (18.6 years, 95%CI 18.5–18.7) and the least number of years free of chronic diseases (9.1 years, 95%CI 8.8–9.4 years) (Table 3). In contrast females of the same age could expect to live the most years free of ADL disability (21.7 years, 95%CI 21.6–22.0) and least free of IADL disability (8.1 years, 95%CI 7.8–8.4).

In terms of gender differences in years free of health problems, patterns were inconsistent. At age 60 females lived significantly more years than males free of chronic diseases (p<0.05), cognitive impairment (p<0.001), depression (p<0.001) and ADL disability (p<0.001) but males lived significantly longer free of IADL disability (p<0.001). At ages 70 and 80 female advantages in years lived free of depression, ADL disability and male advantages in years free of IADL disability remained significant. However, by age 80 males lived significantly longer free of cognitive impairment (p<0.05) (Table 3).

**Fig 1:**
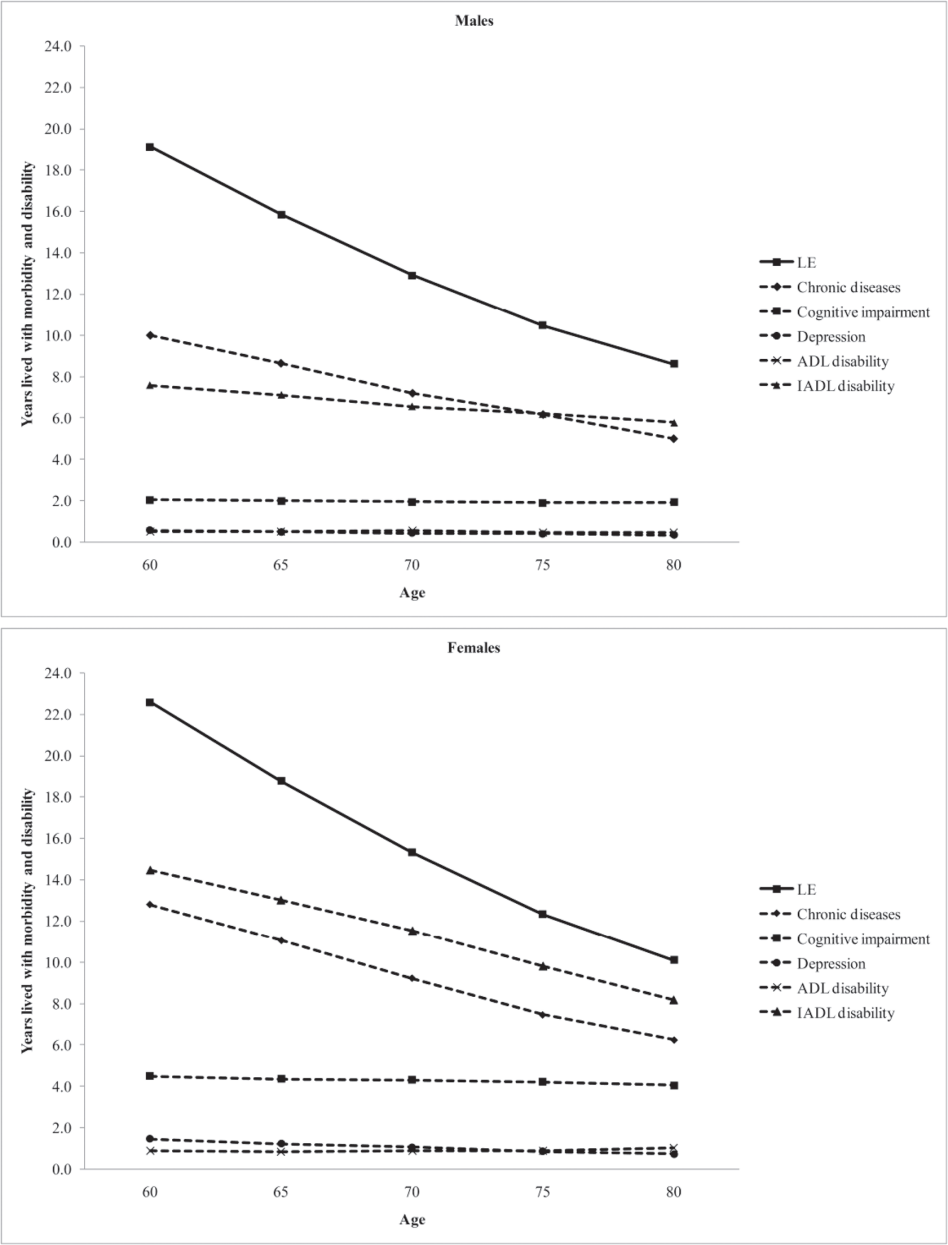
Total life expectancy (LE) and life expectancy spent with morbidity and disability by age and genders in Thailand, 2009.

Fig. 1 illustrates the total life expectancy and life expectancy spent with morbidity and disability measured by each health state by age and gender. Years lived with cognitive impairment, depression and ADL disability were remarkably constant across age though in all cases the number of years lived by females were larger than those for males, and for cognitive impairment, females spent twice as many years with cognitive impairment as males. For chronic diseases years lived with the condition decreased with age similarly to overall life expectancy. Although years lived with IADL disability also decreased with age this was at a slower pace than for overall life expectancy, thought the same patterns for chronic diseases and IADL disability held for males and females.

**Fig 2:**
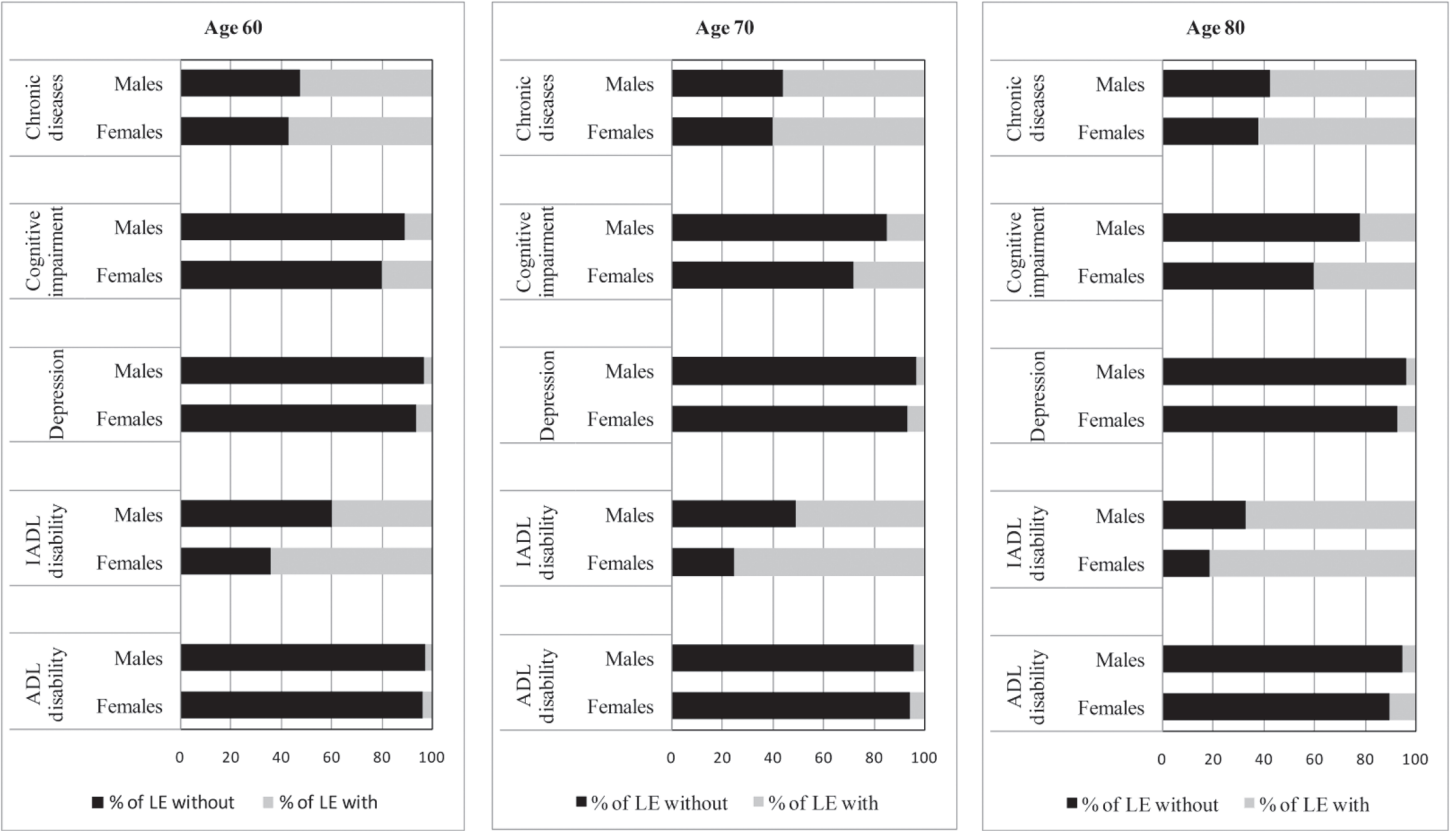
Proportion of total life expectancy (LE) spent without and with morbidity and disability by age and genders in Thailand, 2009.

When health expectancies were expressed as a proportion of total life expectancy, females at all ages, compared to males, spent a greater proportion of their life expectancy with morbidity and disability regardless of the health measure (Fig. 2). For both genders, however, more than 50% of total life expectancy at every age could be expected to be spent with chronic diseases or IADL disability, except for IADL disability in males aged 60 years. In contrast, less than 10% of total life expectancy would be spent with depression or ADL disability.

## Discussion

This is the first study at the national level in Thailand to include multi-dimensions of health (physical and mental) across the disablement process into the calculation of health expectancies. Although the average number of years lived with and without morbidity and disability as measured by the multiple dimensions of health varied as expected, gender differences were not consistent across measures. At age 60, males could expect to live on average the most years free of depression and ADL disability and the least years free of chronic diseases. Females, on the contrary, could expect to live the most years free of ADL disability and the least years free of IADL disability, and they consistently spent more years with all forms of morbidity and disability. Finally, and for both sexes, years lived with cognitive impairment, depression and ADL disability were almost constant with increasing age.

Most health expectancy estimates for Thailand focus on ADL disability, with all using Sullivan’s method and being based on national epidemiological studies in conjunction with national mortality data [13–15]. Between 1997 and 2007, the proportion of life expectancy lived with ADL disability at age 60 was 3–8 percent for males and 4–11 percent for females [13–15]. When compared to values in 2004 [13] and 2007 [15], the prevalence of ADL disability, the absolute number of years lived with disability and the proportion of total life expectancy are slightly lower for males and females at every age. However, it should be noted that the values in 2004 are more comparable to ours as these used earlier waves of NHES whilst the later, 2007, values were based on the national surveys of the elderly where health data was self-reported.

Health expectancy estimates have been available for many Asian countries over the past decade [12]. During the 2000s, studies showed that approximately 6–11 percent of the total life expectancy at age 60 (or 65 years) was lived with ADL disability [27–30], except for Cambodia which had a relatively longer proportion [31] and almost all these studies are methodologically comparable to ours in terms of data type and calculation method. Compared to other Asian populations, Thai older people appear to have a better quality of life in terms of performance of self-care activities. However, to compare our findings with those of other studies, both within the country and internationally, issues of disability definitions and survey design should be taken into consideration. ADL items selected in the definitions vary across studies which potentially leads to different severity levels of disability. In addition, the health surveys used in most previous studies are based on self-report while ours employed mixed methods including a health examination, which might therefore be subject to a different form of bias (e.g. from refusal to participate in those with severe illness or mobility problems) rather than under or over reporting of abilities.

Few studies in Asia concentrate on IADL disability and chronic diseases although ours showed that older people in Thailand could expect to spend the most years with these conditions (10 and 13 years with chronic diseases, and 8 and 14 years with IADL disability for males and females aged 60, respectively). A study in Japan examining IADL disability by using longitudinal data showed that 13% of males’ and 18% of females’ life expectancy at age 65 years was spent with disability in IADL but not ADL [29]. IADL performance might not solely indicate the underlying health of a population since it is also influenced by environment and role expectation. The better quality of life in IADL performance in Japan, compared to the findings presented here, might therefore partly be explained by its more age-friendly environment, e.g. accessibility to public transports. With regard to chronic diseases, there has been little harmonization in measurements, which makes comparison difficult. A study in Singapore calculated disease-specific health expectancies, e.g. years with and without heart disease, cancer, diabetes, and hypertension [30]. In contrast, studies in China and Hong Kong, as ours, combined a number of diseases which required long-term medical follow-up [27,28].

Direct comparison with studies of mental health expectancy, all of which have been conducted in Western countries, is difficult, primarily due to characteristics of the data sources in terms of protocols, methods of data collection, and measurement tools [6]. The focus therefore is on how years lived with mental disorders change across age for our study compared to others. The most noticeable finding, consistently shown across studies including ours, is that years spent with cognitive impairment appears relatively constant across old age [32–34]. For depression, a study in European countries provided estimates of depression-free life expectancy in early- to mid-life (age 25 to 55) and found that years with depression slightly decreased with age both in absolute values and as relative to the total life expectancy [35]. This appears different to the pattern in older age in our study and this might link with changes that often come in later life, e.g. retirements, medical problems, etc. In terms of service provision, our findings imply that on average three years (both genders combined) of care for the cognitively impaired has to be provided for every person aged 60 years and over during their lifetime.

Our findings that females live more years with all forms of morbidity and disability suggest that females’ longer lives are not necessarily healthy ones and confirms other Asian studies which have consistently shown that females spend a greater proportion of their life expectancy lived with chronic diseases [28,30] and disability in terms of usual activities [36], mobility [37], and ADL performance [13–15,28,30,31,36]. According to Oksuzyan et al. [38], this phenomenon is called the female-male health-survival paradox and reasons for it are still not fully understood. Multiple causes have been proposed, including hormonal, autoimmune, and genetic differences between the genders, and gender differences in lifestyle factors (such as risk-taking behaviour) and health behaviours (such as help-seeking behaviour, compliance with medical treatment) might also contribute [38,39]. In the Thai context, as in some other countries, worse health in females might be partly linked to education since traditionally boys were more often favoured for schooling than girls, which is more apparent in the old age cohorts [40]. In addition, gender gaps in IADL disability might be influenced by gender differences in role expectation in some activities, e.g. housework. According to the model of disablement process and the more recent ICF, disability is not inherent in a person [9,10]. Instead, it denotes a relationship between personal capability and the demand of environment. Further, gender differences in morbidity may be due to biases in reporting health as gender stereotypes and social roles make it culturally more acceptable for females to have and report illness and health problems [38,39]. However, in our study, there is likely to be less gender bias in reporting since some measures, specifically chronic diseases and cognitive impairment, were validated by physical examination, or laboratory or screening tests.

This study has both limitations and strengths. First, some limitations lie in its use of Sullivan’s method. This method uses the observed prevalence which is a result of the past incidence and mortality experience of each cohort in the survey rather than the current incidence rates [21,41]. So, it might not be able to reflect current morbidity or disability patterns. Despite this, Sullivan’s method is the most often used because it requires cross-sectional data and period life tables that are widely available [21,41], and it has been shown to provide unbiased estimates of health expectancy if the transitions are stable and smooth over time [41]. A second limitation, common to other health expectancy estimates, is that institutionalized people were not included because there are no public data available by age and sex. Overall there are approximately 7,000 institutionalized older adults in 2010 (combining those in nursing homes and prisons), which accounts for less than 0.1 percent of total older population [42], thus this is unlikely to have a major effect on our estimates. Finally, and as previously mentioned, the health examination survey may have resulted in an underestimate of the prevalence of morbidity or disability since people with severe illness or mobility problems (e.g. hemiplegic conditions) may not have been able to participate. Though the alternative of prevalence estimates based on self-report of disability may have other biases, such non-response due to ill-health may be a reason for the lower prevalence of ADL disability in participants aged 75–79 years, compared to those aged 70–74. Nevertheless, the use of mixed methods for ascertainment of health status in the NHES is also a strength since physical examinations, and functional and laboratory tests, enable the ascertainment of pathologies or impairments of which individuals themselves are unaware and therefore unable to self-report. In addition, the use of mixed methods might also resolve some of the issues of differential reporting of health between socio-economic groups or individuals with and without cognitive impairment [43].

In conclusion, this study adds knowledge of gender differences in healthy life expectancy in the older Thai population using a wider spectrum of health than previously which provides information to inform different groups of policy audiences [17]. For instance, health expectancy measured by chronic physical diseases is important for informing the type and amount of public health resources required for prevention and care whilst the number of years lived with ADL disability is information primarily needed by social and health policy-makers who have responsibility for the provision of long-term care. In addition, health expectancy covering the spectrum of the disablement process can inform the progression of different of health states, for example it is informative about the average time spent with chronic physical diseases or cognitive impairment before developing ADL disability, a period which is potentially significant for secondary and tertiary prevention.
